# Development and Validation of a Robust Immune-Related Prognostic Signature for Gastric Cancer

**DOI:** 10.1155/2021/5554342

**Published:** 2021-04-30

**Authors:** Junyu Huo, Liqun Wu, Yunjin Zang

**Affiliations:** ^1^Liver Disease Center, The Affiliated Hospital of Qingdao University, No. 59 Haier Road, Qingdao 266003, China; ^2^Qingdao University, No. 308 Ningxia Road, Qingdao 266071, China

## Abstract

**Background:**

An increasing number of reports have found that immune-related genes (IRGs) have a significant impact on the prognosis of a variety of cancers, but the prognostic value of IRGs in gastric cancer (GC) has not been fully elucidated.

**Methods:**

Univariate Cox regression analysis was adopted for the identification of prognostic IRGs in three independent cohorts (GSE62254, *n* = 300; GSE15459, *n* = 191; and GSE26901, *n* = 109). After obtaining the intersecting prognostic genes, the three independent cohorts were merged into a training cohort (*n* = 600) to establish a prognostic model. The risk score was determined using multivariate Cox and LASSO regression analyses. Patients were classified into low-risk and high-risk groups according to the median risk score. The risk score performance was validated externally in the three independent cohorts (GSE26253, *n* = 432; GSE84437, *n* = 431; and TCGA, *n* = 336). Immune cell infiltration (ICI) was quantified by the CIBERSORT method.

**Results:**

A risk score comprising nine genes showed high accuracy for the prediction of the overall survival (OS) of patients with GC in the training cohort (AUC > 0.7). The risk of death was found to have a positive correlation with the risk score. The univariate and multivariate Cox regression analyses revealed that the risk score was an independent indicator of the prognosis of patients with GC (*p* < 0.001). External validation confirmed the universal applicability of the risk score. The low-risk group presented a lower infiltration level of M2 macrophages than the high-risk group (*p* < 0.001), and the prognosis of patients with GC with a higher infiltration level of M2 macrophages was poor (*p* = 0.011). According to clinical correlation analysis, compared with patients with the diffuse and mixed type of GC, those with the Lauren classification intestinal GC type had a significantly lower risk score (*p* = 0.00085). The patients' risk score increased with the progression of the clinicopathological stage.

**Conclusion:**

In this study, we constructed and validated a robust prognostic signature for GC, which may help improve the prognostic assessment system and treatment strategy for GC.

## 1. Background

Gastric cancer (GC) originates from the most superficial mucosal epithelial cells of the gastric wall [1]. GC development is a progressive process involving many factors and steps, such as genetic factors, *Helicobacter pylori* infections, dietary or environmental factors, and precancerous states [2, 3]. The latest statistical results showed that GC ranked first among the malignant tumors of the digestive system, representing a serious threat to human health [4, 5]. Although the overall survival (OS) of GC has been significantly increased in recent years due to the diversification of treatments, such as targeted therapy, angiogenic therapy, and immunotherapy [6], there is still much room for progress in improving the prognosis of GC with a surgery-based comprehensive treatment strategy.

Genetics were found to play a crucial role in the molecular mechanism responsible for GC occurrence and growth [7]. At present, many genes related to GC have been found. Some of these genes exist in the cell genome in the form of oncogenes, encode proteins required for cell growth, and drive carcinogenesis when they are activated by point mutation, translocation, and amplification [8]. Some of the genes are tumor suppressors, which play a negative regulatory role in controlling cell growth, proliferation, and differentiation [9]. Finally, some of the genes affect the biological process of GC by altering the drug resistance of the cancer cells [10]. Therefore, a full understanding of the function of genes in the progression of GC is expected to enrich our understanding of the therapeutic targets of GC.

A growing number of reports have revealed that immune-related genes (IRGs) have a significant impact on the prognosis of a variety of cancers [11–14]. However, the prognostic value of IRGs for GC has not been fully elucidated. In this study, we integrated and analyzed high-throughput sequencing data from public databases and provided a robust prognostic signature for GC based on nine IRGs. The predictive power of the prognostic signature was confirmed by internal and external validation. Meanwhile, we explored the prognostic mechanism of the signature by combining quantitative analysis regarding immune cell infiltration (ICI) with correlation analysis regarding clinical features.

## 2. Materials and Methods

### 2.1. Data Collection

The Cancer Genome Atlas (https://portal.gdc.cancer.gov/) and the Gene Expression Omnibus (GEO) databases (https://www.ncbi.nlm.nih.gov/geo/) were used for the collection of gene expression profiles and clinical data. The data utilized in this study were obtained from public databases. We complied with the access rules of TCGA and GEO databases during the process of data collection, and it was unnecessary to obtain approval from the local ethics committee.

### 2.2. Identification of Prognostic Immune-Related Genes

We extracted data on the immune-related genes of three independent cohorts (GSE62254, *n* = 300; GSE15459, *n* = 191; and GSE26901, *n* = 109) according to the immune-related gene list acquired from the ImmPort database (https://immport.niaid.nih.gov). Next, we set *p* < 0.05 as the screening criterion and screened the prognosis-related genes by univariate Cox regression analysis in the three independent cohorts.

### 2.3. Cluster Analysis of the Intersection Prognostic Genes

We merged GSEE62254, GSE15459, and GSE26901 into a training cohort (*n* = 600), and the batch effects between different datasets were removed by the “combat” function in the R package “sva.” We conducted cluster analysis based on the R package “consensusclusterplus” to further analyze the prognostic value of overlapping prognostic genes.

### 2.4. Development and Validation of a Prognostic Signature

Based on the least absolute shrinkage and selection operator (LASSO) algorithm, we removed the overfitting between prognosis-related genes by 10-fold cross-validation for penalty parameter tuning and kept genes that had nonzero regression coefficients for later multivariate Cox regression analyses [15, 16]. Each gene's regression coefficient obtained from such analysis was multiplied by its expression level, thus resulting in the risk score [16, 17]. Based on the median score, we divided patients with GC into low- and high-risk groups. The LASSO regression analysis was performed with the “glmnet” R package, where the time-dependent ROC curve was plotted with “survivalROC” and the Kaplan–Meier survival curve was generated with “survminer.” This analysis facilitated the evaluation of the risk score's performance [14]. In addition, with the “rms” R package, the prognostic signature was visualized in the form of a nomogram, and the calibration curve was used to evaluate the nomogram's predictive accuracy. The prognostic signature's performance was externally validated in the three independent cohorts (GSE26253, *n* = 432; GSE84437, *n* = 431; and TCGA, *n* = 336).

### 2.5. Analysis of the Relationship between Immune Cell Infiltration and the Prognostic Signature

Immune cell infiltration (ICI) was quantified by the CIBERSORT method, and the sample filtration threshold was *p* < 0.05. We used the Wilcox test to compare the ICI difference between different risk groups, where the statistical significance was expressed as *p* < 0.05.

### 2.6. Analysis of the Relationship between Clinical Characteristics and Prognostic Signature

We analyzed the prognostic differences among patients with GC with different clinical features and conducted a comparison of the low- and high-risk groups' clinical characteristics. In addition, we assessed the distribution of risk scores of patients with different clinical features.

### 2.7. Comparison of the Immune Response in Different Risk Groups

To reveal the potential immune signature of the prognostic model, we selected 93 immune-related gene sets from “c5.all.v7.3.symbols.gmt” (downloaded from http://www.gsea-msigdb.org/gsea) and conducted a gene set enrichment analysis (GSEA) of the different risk groups. In general, the higher the enrichment score (ES), the stronger the activity of the pathway.

### 2.8. Analysis of the Relationship between Tumor Microenvironment and Risk Score

The stromal cells and immune cells were main components in the tumor microenvironment (TME). The ESTIMATE (Estimation of STromal and Immune cells in MAlignant Tumor tissues using Expression data) algorithm could quantify the immune and stromal components in the tumor microenvironment (TME) by analyzing the specific gene expression characteristics of immune and stromal cells, which was employed to calculate the Stromal Score (captures the presence of stroma in tumor tissue), Immune Score (represents the infiltration of immune cells in tumor tissue), and ESTIMATE Score (equaled the sum of Stromal Score and Immune Score) [18]. We assigned patients into high- and low-level groups by comparison to the median value of the scores and performed Kaplan–Meier survival analysis to investigate the relationship between the scores and the GC prognosis. The Spearman correlation test was used to analyze the association of the risk score with the TME scores.

## 3. Results

### 3.1. Patients with GC Had Different Clinical Outcomes with Different Gene Clusters

**Figure 1:**
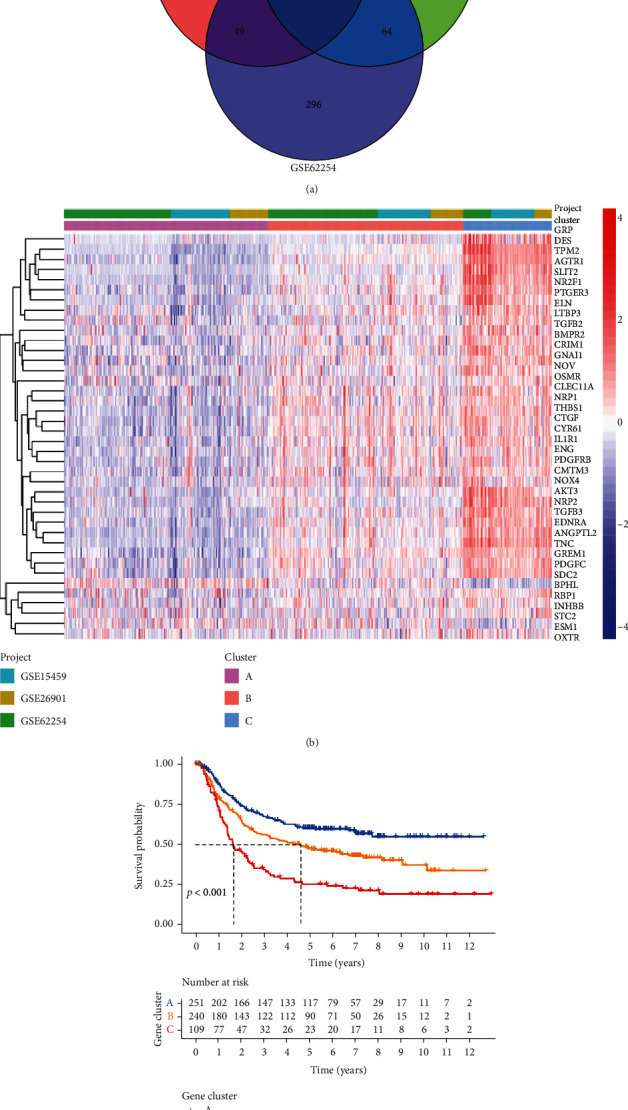
Gene cluster analysis. (a) Venn plot of the 45 intersecting prognosis-related genes. (b) Heatmap of gene clusters. (c) Kaplan–Meier survival analysis regarding gene clusters and OS in the training cohort.

A total of 45 overlapping prognosis-related genes were identified in the three independent cohorts (GSE62254, GSE15459, and GSE26901) (Figure 1(a), Table 1). The 600 patients with GC were divided into three subtypes based on the 45 gene cluster analysis (Figure 1(b)). There were significant differences in the OS among patients with different subtypes of GC (Figure 1(c)), which preliminarily implied the significant impact of the expression of the 45 genes on the prognosis of GC.

### 3.2. A Nine-Gene Prognostic Signature Was Established in the Training Cohort

**Figure 2:**
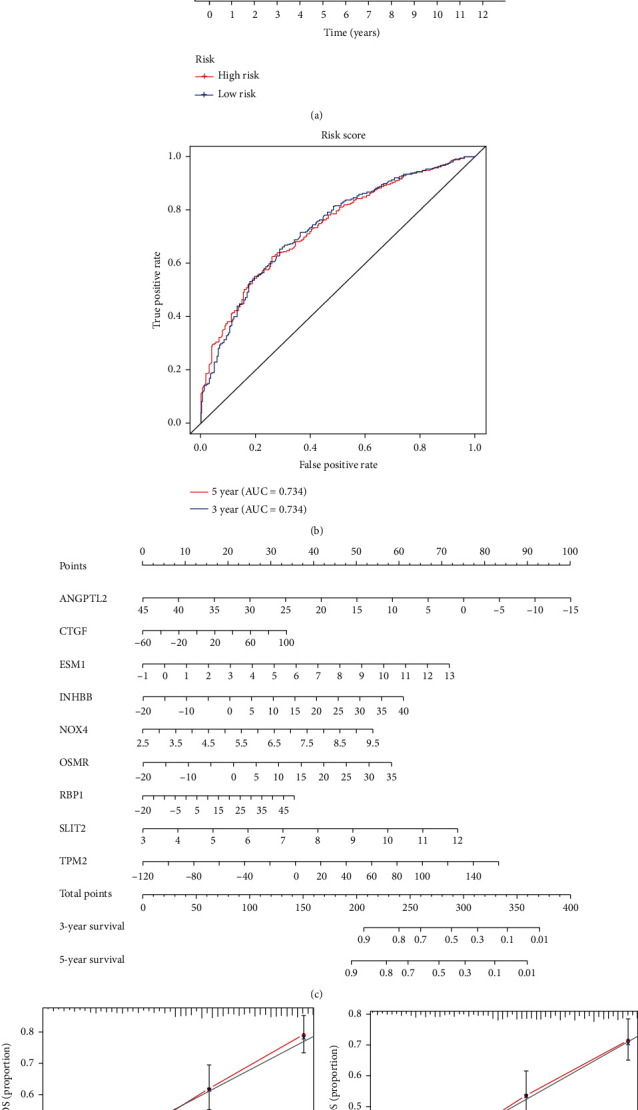
Construction of a nine-gene prognostic signature. (a, b) Kaplan–Meier survival analysis and time-dependent ROC analysis regarding risk score and OS in the training cohort. (c, d) Prognostic signature visualized as a nomogram and tested by the calibration curve.

**Figure 3:**
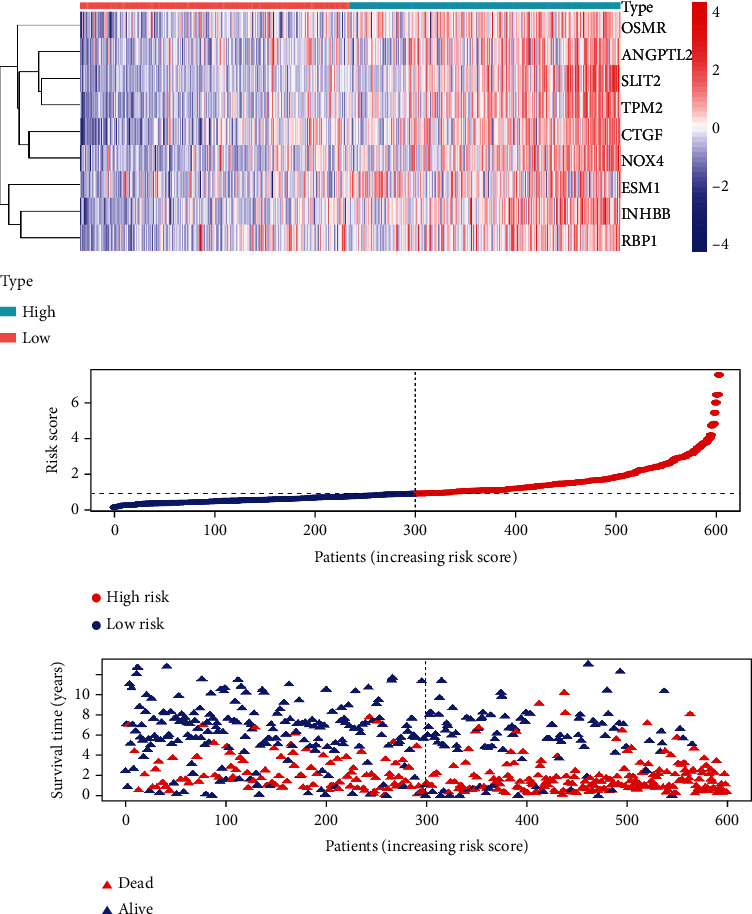
Heatmap, risk score distribution, and survival status of patients in the training cohort.

The risk score established by LASSO regression and multivariate Cox regression analyses was calculated as follows: ANGPTL2∗−0.03827 + CTGF∗0.004835 + ESM1∗0.117437 + INHBB∗0.0233 + NOX4∗0.17667 + OSMR∗0.02425 + RBP1∗0.011599 + SLIT2∗0.18834 + TPM2∗0.006843. The 600 patients with GC were divided into low- and high-risk groups according to the median risk score (0.966). The high-risk group had a lower OS than the low-risk group (Figure 2(a)). The area under the curve (AUC) values for the risk score predicting 3- and 5-year OS were 0.734 and 0.734, respectively (Figure 2(b)). The calibration curve also revealed the consistency between the actual OS and the signature-predicted OS (Figures 2(c) and 2(d)). The high-risk group presented upregulated expression levels of the 9 genes, and the patients' risk of death increased as the risk score increased (Figure 3). Univariate and multivariate Cox regression identified the risk score as an independent indicator for the prognosis of patients with GC (Table 2), and the detailed clinical information is displayed in supplement material 1.

### 3.3. The Prognostic Signature Showed High Accuracy for the Prediction of the OS of Patients with GC in the GSE62254, GSE15459, and GSE26901 Cohorts

**Figure 4:**
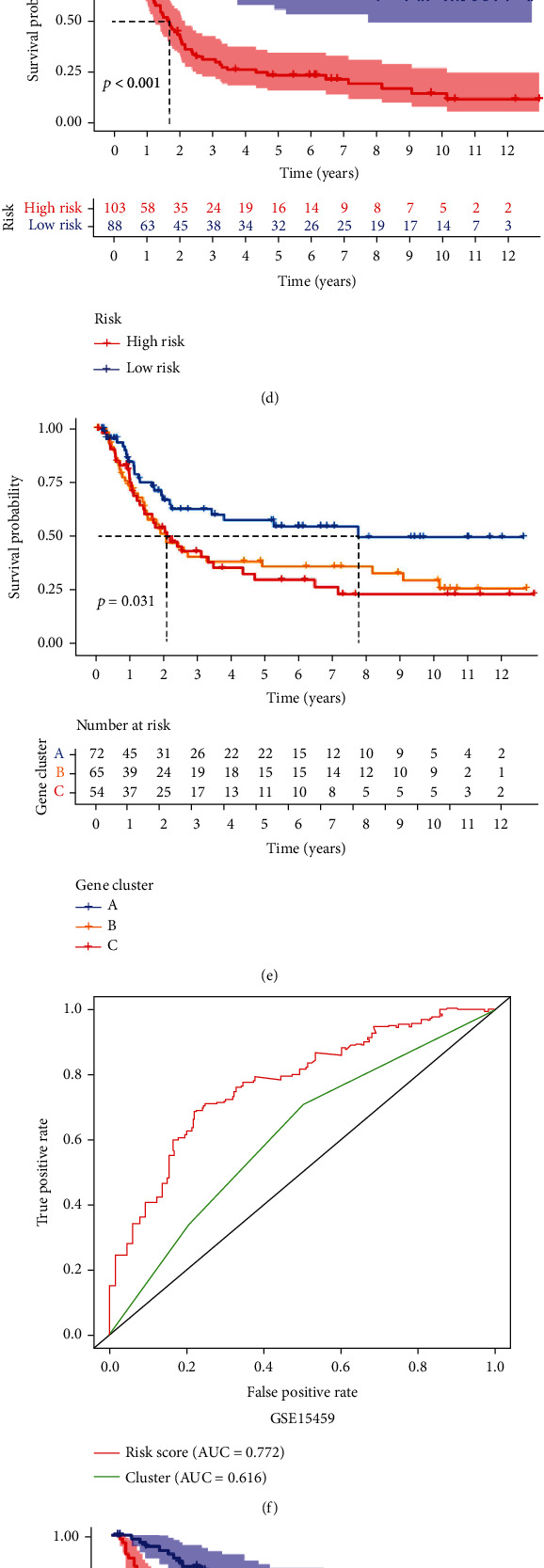
Internal validation of the prognostic signature. (a–c) Kaplan–Meier survival analysis and the time-dependent ROC analysis of the risk score and gene cluster for predicting OS in the GSE62254 cohort. (d–f) Kaplan–Meier survival analysis and the time-dependent ROC analysis of the risk score and gene cluster for predicting OS in the GSE15459 cohort. (g–i) Kaplan–Meier survival analysis and the time-dependent ROC analysis of the risk score and gene cluster for predicting OS in the GSE26901 cohort.

The high-risk group had a significantly lower OS than the low-risk group in each independent cohort (Figures 4(a), 4(d), and 4(g)). The accuracy of the risk score was higher than that of the gene cluster for the stratification of the prognostic risk of patients with GC (Figures 4(b), 4(c), 4(e), 4(f), 4(h), and 4(i)).

### 3.4. The Prognostic Signature Showed Robustness through External Validation

**Figure 5:**
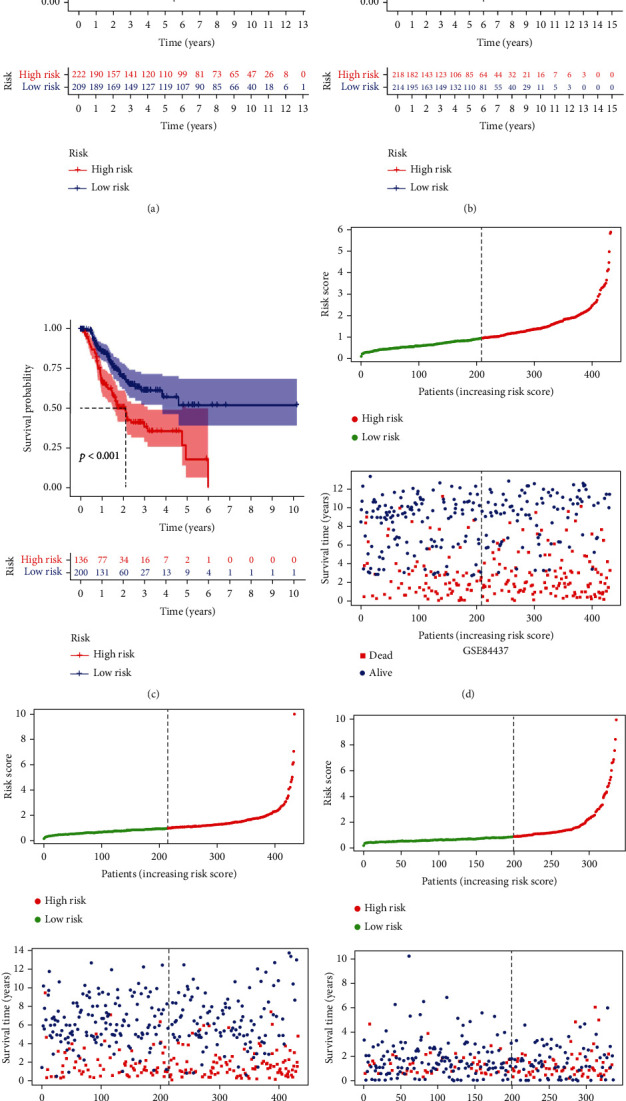
External validation of the prognostic signature. (a–c) Kaplan–Meier survival analysis of the prognostic signature for predicting OS in the GSE84437, GSE26253, and TCGA cohorts. (d–f) The risk score distribution and the survival status of patients in the GSE84437, GSE26253, and TCGA cohorts.

Consistent with the results of internal validation, compared with the low-risk group in each independent cohort, the high-risk group had significantly reduced OS and recurrence-free survival (RFS) (Figures 5(a)–5(c)). As the risk score increased, so did the risk for death and recurrence (Figures 5(d)–5(f)).

### 3.5. The High- and Low-Risk Groups Exhibited Differences in Immune Cell Infiltration

**Figure 6:**
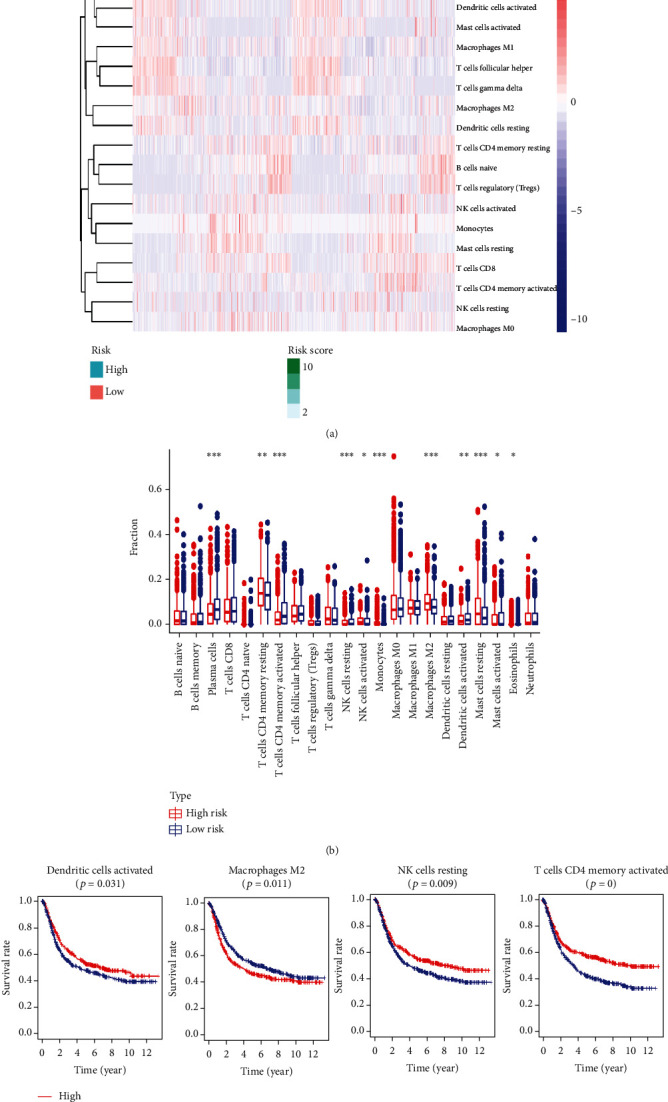
The immune cell infiltration landscape of all included samples. (a, b) Heatmap and the boxplot showing the difference in immune cell infiltration in different risk groups. (c) Kaplan–Meier survival analysis regarding immune cell infiltration and OS.

The tissues from patients with high-risk GC exhibited a lower infiltration level of resting NK cells, activated memory CD4 T cells, and activated dendritic cells. However, the tissues from patients with low-risk GC had a lower infiltration level of M2 macrophages than the high-risk group (Figures 6(a) and 6(b)). The prognosis of patients with GC with a higher infiltration level of M2 macrophages was poor; patients with a higher infiltration level of resting NK cells, activated memory CD4 T cells, and activated dendritic cells had a better prognosis (Figure 6(c)).

### 3.6. The Risk Score Was Closely Related to the Clinicopathological Features

**Figure 7:**
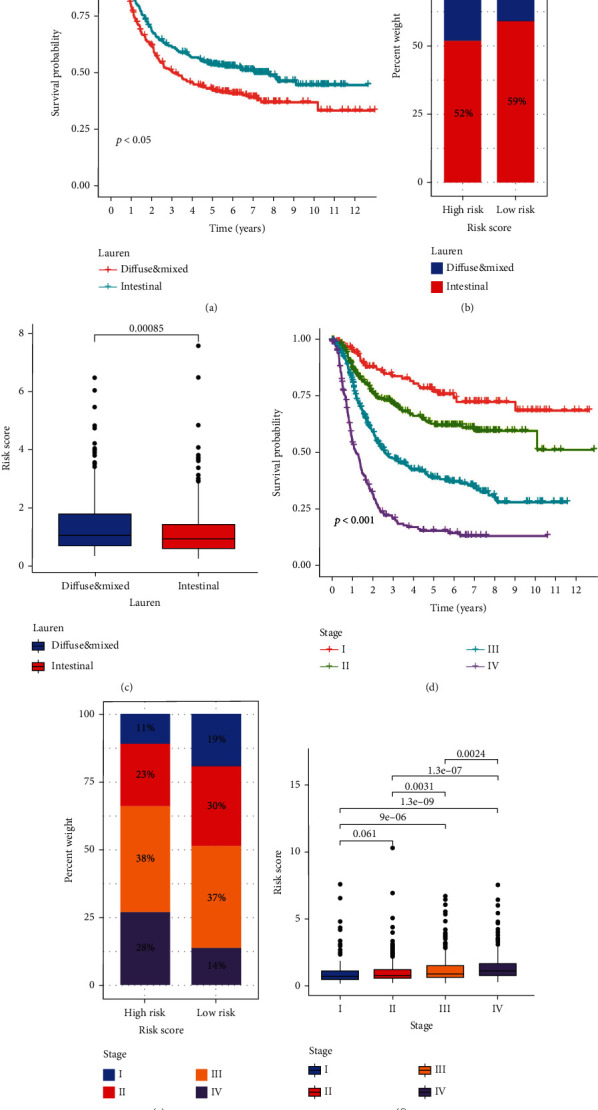
The correlation analysis between the risk score and clinical characteristics. (a) The Kaplan–Meier survival analysis regarding Lauren classification and OS. (b) Barplot of proportions of different Lauren classification subtypes in high- and low-risk groups. (c) Boxplot of the risk score difference in different Lauren classification subtypes. (d) Kaplan–Meier survival analysis regarding stage and OS. (e) Barplot of proportions of different stages in high- and low-risk groups. (f) Boxplot of the risk score difference in different stages.

The prognosis of patients with the Lauren classification intestinal type of GC was better than that of patients with the diffuse type and mixed type (Figure 7(a)). The high-risk group had a lower proportion of patients with the intestinal type than the low-risk group (Figure 7(b)); patients with the intestinal type had a significantly lower risk score compared to those with the diffuse type and mixed type (Figure 7(c)). The OS of patients decreased significantly with increasing disease stage (Figure 7(d)). The high-risk group had a significantly lower proportion of early-stage patients than the low-risk group, but almost double the number of patients in stage IV (Figure 7(e)), in line with the positive correlation between the risk score and disease stage (Figure 7(f)).

### 3.7. The Immune Response of the Low-Risk Group Was Stronger Than That of the High-Risk Group

**Figure 8:**
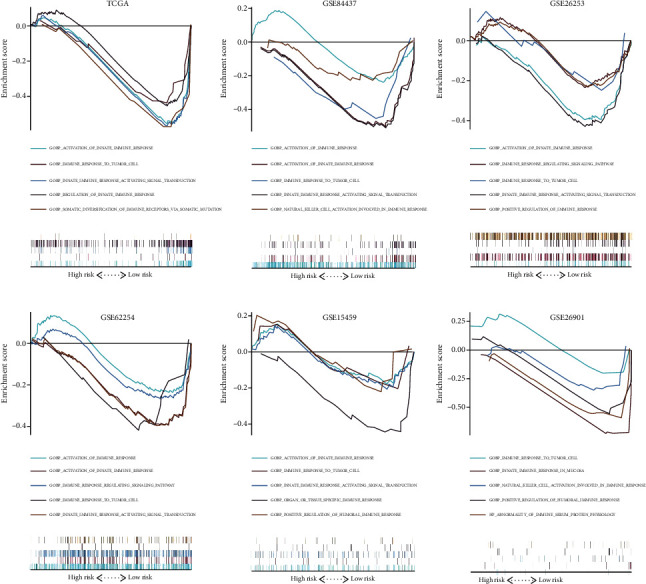
Gene set enrichment analysis for immune-related pathways in different risk groups.

In the six independent cohorts, we found that immune response-related pathway ESs in the high-risk group were lower than those in the low-risk group, including the activation of the innate immune response, regulation of the innate immune response, and immune response to tumor cells (Figure 8).

### 3.8. The Risk Score Exhibited a Significant Positive Correlation with the Stromal Score

**Figure 9:**
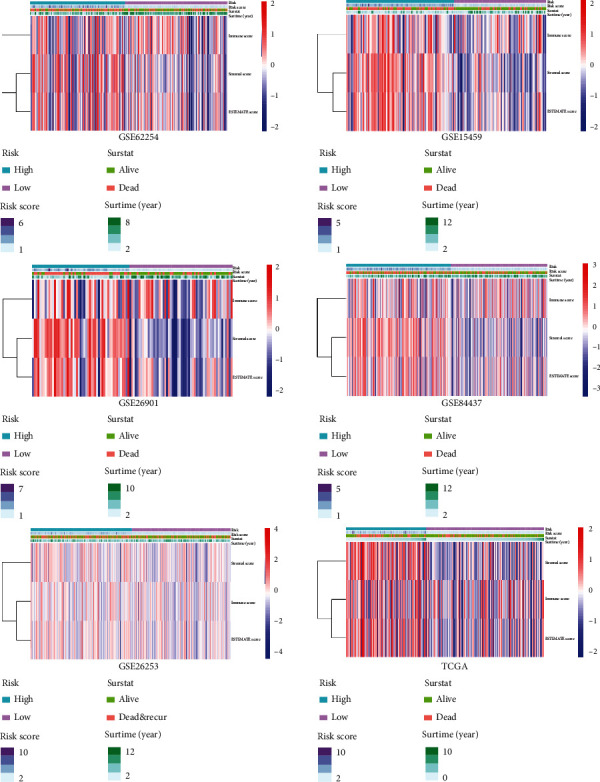
The presentation of ESTIMATE calculation results.

**Figure 10:**
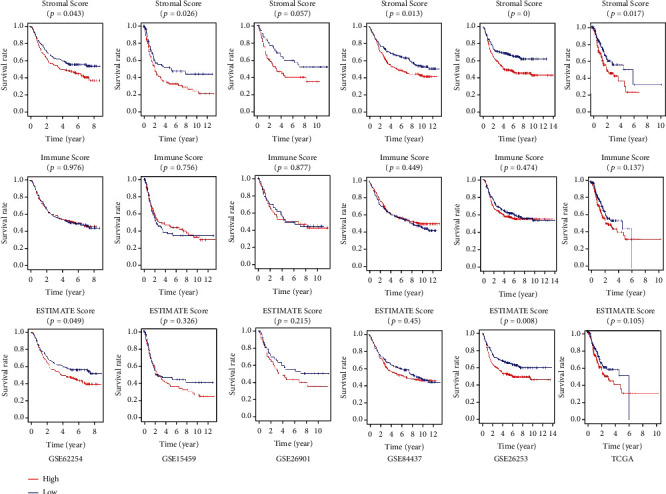
The Kaplan–Meier survival analysis of Stromal Score, Immune Score, and ESTIMATE Score in the six independent cohorts.

**Figure 11:**
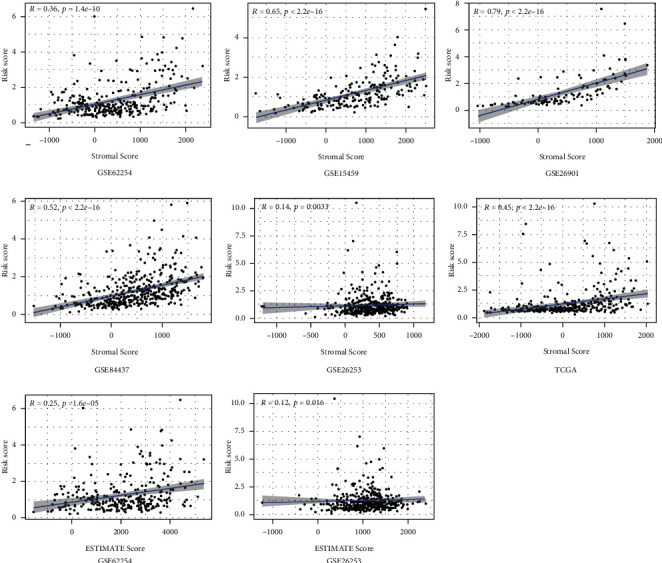
The association between risk score and the Stromal Score, Immune Score, and ESTIMATE Score.

We calculated the Stromal Score, Immune Score, and ESTIMATE Score of the GC samples with the ESTIMATE algorithm to facilitate the quantification of immune and stromal components in the TME in the six independent cohorts (Figure 9). Compared with the GC patients with lower Stromal Score, we found the GC patients with higher Stromal Score had a significantly decreased OS and RFS in the six independent cohorts (Figure 10). The Spearman correlation analysis suggested that the risk score and the Stromal Score were significantly positively correlated with each other (Figure 11). These results may indicate that the higher the risk score, the higher the content of neovascularization, endothelial cells, and mesenchymal stem cells around the tumor, facilitating the formation of TME to promote tumor proliferation and metastasis.

## 4. Discussion

Gastric cancer (GC) is a prevailing malignant tumor and, second to lung cancer, has the highest incidence and mortality rates in the world [19]. Most patients with GC have advanced-stage disease when they are diagnosed, which leads to poor prognosis after surgery [20]. However, it is difficult to accurately evaluate the prognosis of GC, namely, the risk for recurrence and metastasis and likely survival time, using conventional TNM staging [21]. Therefore, identifying an effective prognosis evaluation scheme for GC remains a relevant and challenging research topic.

Normally, the immune system can recognize and remove tumor cells from the tumor microenvironment [22]. However, to survive and grow, tumors use multiple mechanisms to develop immune tolerance and prevent the immune system from effectively recognizing and killing tumor cells [23]. Immune-related genes are crucial for the regulation of tumor progression and the human immune response. It is particularly important to identify how immune-related genes affect the prognosis of GC to develop a prognostic evaluation scheme for GC.

We identified 45 common prognostic immune-related genes in three different independent cohorts. According to the cluster analysis of the 45 overlapping genes, there were significant differences in the OS among different patients with different subtypes of GC. We integrated the GSE62254, GSE15459, and GSE26901 cohorts into a training cohort, screened the above 45 intersecting genes again by LASSO and multivariate Cox regression analyses, and finally created a risk score comprising 9 genes. Compared to gene cluster analysis, the risk score reduced the number of genes that need to be sequenced, saved costs, and had higher accuracy. The risk score showed universal applicability via internal and external validation. The risk score is also an independent prognostic indicator of the risk for death and recurrence in patients with GC. Additionally, we found that the risk score showed better performance than that of a previous study [24] in predicting RFS and OS in patients with GC (supplemental material 2), especially for the GSE62254 [25], GSE26253 [26], and GSE26901 [26] cohorts. Considering that the above three datasets mainly originated from Asian populations, we speculated that our model is superior in evaluating the prognosis of Asian patients with GC. To test our hypothesis, we observed the survival of Asian patients in TCGA dataset. Although only 69 patients were included, we found that the OS of 24 high-risk patients was significantly (*p* < 0.001) lower than that of 45 low-risk patients, and the time-dependent ROC analysis also demonstrated that the risk score had high accuracy (supplement material 3). Therefore, this evidence suggests that our model is a promising prognostic predictor for Asian patients with GC.

Next, we assessed the potential mechanism of the risk score with a focus on immune cell infiltration (ICI) and clinicopathology. The high-risk group presented a significant increase in the infiltration level of M2 macrophages, and M2 macrophages were a risk factor for GC prognosis. It is generally believed that M2 macrophages play a leading role in cancer progression and metastasis [27]. In addition, we identified three kinds of immune cells, resting NK cells, activated memory CD4 T cells, and activated dendritic cells, which were favorable for the prognosis of GC. They all showed a higher infiltration degree in the low-risk group, which has rarely been reported before. In terms of clinical features, we focused on the correlation between the risk score and the Lauren classification and clinicopathological stage. Lauren classification is a method that combines cell morphology and histochemistry to classify GC cells [28]. This method divided GC into the intestinal type and the diffuse type. Previous studies have shown that intestinal-type GC has a higher degree of differentiation, is more common in the elderly, has a low degree of malignancy, and has a better prognosis [29], while diffuse-type GC is often undifferentiated, has a poor prognosis, and is difficult to treat [30]. We found that the risk score of intestinal-type GC was low, and the prognosis was good, which was consistent with previous reports. In clinical practice, the most popular prognostic evaluation system for GC is still TNM staging [31]. We found that the risk score increased with increasing stage, indicating that the risk score was positively correlated with disease stage but negatively correlated with prognosis. In addition, the GSEA results showed that the activation of the immune response in the high-risk group was weaker than that of the low-risk group, which indicated that the poor prognosis of the high-risk group may be related to immunosuppression. In terms of TME, the GC patients with higher Stromal Score had decreased OS and RFS, so targeted therapy of stromal cells in TME may have a positive effect on the prognosis of GC patients. Considering the risk score exhibited a significantly positively correlation with the Stromal Score, we speculated that the higher risk score may facilitate the formation of TME to promote tumor proliferation and metastasis; the nine genes in the signature may be a promising target for the treatment of GC.

Therefore, compared with previous studies [32–34], this research has the following advantages. First, a total of 1799 GC patients from six independent datasets were included in this work, which was one of the largest GC prognostic model development projects to our knowledge. Second, the proposed signature has robustness in the prediction of the prognosis of GC through internal and external validation, especially for Asian GC patients. In view of the fact that the Asian region has the highest incidence and death of GC all over the world [35], the proposed prognostic model may receive increasing attention in the future. Third, we found the close correlation between the risk score and the stromal cells by investigating the TME, which was also a novel finding of this paper, considering similar reports were rare before, which may provide new clues for targeted therapy of stromal cells in GC.

Tumor cell-derived angiopoietin-like protein 2 (ANGPTL2) activates tumor cell motility, invasiveness, and epithelial-mesenchymal transition to accelerate tumor metastasis in an autocrine/paracrine manner [36]. Recently, novel research indicated that stroma-derived ANGPTL2 could drive the production of immune-stimulated macrophages through the NF-*κ*B pathway and accelerate the activation of CD4 T helper 1 (Th1) cells to play an antitumor role [37]. Increasing evidence has shown that connective tissue growth factor (CTGF) is a multifunctional signal regulator that promotes the occurrence, progression, and metastasis of cancer by regulating epithelial-mesenchymal transition (EMT), invasion, migration, cell proliferation, and drug resistance [38]. Esophageal cancer (ESCA) was accompanied by high upregulation of ESM1, which could be partly explained by cell proliferation and migration and the regulation of the Janus kinase (JAK) signaling pathway [39]. As a protein-coding gene, inhibin subunit *β* B (INHBB) is involved in the synthesis of transforming growth factor-*β* (TGF-*β*) family members. Yuan et al. found that overexpression of INHBB largely contributed to macrophage infiltration and impeded the infiltration of memory T cells, mast cells, and dendritic cells in colorectal cancer and was correlated with worse OS and DFS [40]. According to Chen et al. [41], the growth and metastasis of an orthotopic hepatocellular carcinoma (HCC) tumor were promoted by the Sox9/INHBB axis, which activated peritumoral hepatic stellate cells (HSCs). NADPH oxidase 4 (NOX4) is a key regulator of reactive oxygen species production [42]. Ford et al. [43] reported that NOX4 is essential for maintaining the immunosuppressed tumor-associated fibroblast (CAF) phenotype in tumors. NOX4 inhibition could effectively overcome the resistance of CAF-mediated immunotherapy and improve the prognosis of many cancers. Sharanek et al. [44] found that the cytokine receptor for oncostatin M (OSMR) regulated the proliferation of glioblastoma by regulating oxidative phosphorylation to resist ionizing radiation. Retinol binding protein 1 (RBP1) plays a role in many physiological functions, such as regulating retinol transport and metabolism. Gao et al. [45] demonstrated that the overexpression of RBP1 promoted the growth, invasion, and migration of oral squamous cell carcinoma (OSCC) cells and that silencing RBP1 inhibited tumor formation in xenograft mice. SLIT2 glycoprotein has been described to regulate the inflammatory response and participate in autoimmune diseases [46]. The endothelial-derived SLIT2 protein, together with its receptor ROBO1, drove cancer cells to migrate and infiltrate into endothelial tissue. In mouse models of breast cancer and lung cancer, the deletion of SLIT2 inhibited metastasis. In contrast, when tumoral SLIT2 was blocked, metastatic progression was enhanced [47]. The results of Zhou et al.'s single-cell multiomics sequencing revealed that TPM2 was a fibroblast-specific biomarker associated with poor prognosis in colorectal cancer [48].

In this study, we systematically analyzed the prognostic value of immune-related genes in GC by integrating multiple sequencing datasets and clinical information. A risk score comprising 9 genes showed good performance in forecasting the prognosis of GC, and internal and external validation further confirmed its robustness. Since this study is retrospective, prospective clinical trials are still necessary in the future. The specific function of the nine genes in the progression of GC is still unknown and needs to be verified via further experiments.

## 5. Conclusion

In this study, we constructed and validated a robust prognostic signature for GC by integrating multiple sequencing datasets and clinical information, which may help improve the prognostic assessment system and treatment strategy for GC.

## Figures and Tables

**Table 1:** The 45 intersection prognosis-related genes identified by univariate Cox regression analysis in the training cohort.

Gene list	Category	HR	HR.95L	HR.95H
AGTR1	Cytokine_receptors	1.334317	1.217829	1.461947
AKT3	BCRSignalingPathway	1.761968	1.444244	2.149589
ANGPTL2	Cytokine_receptors	1.344887	1.192773	1.5164
BMPR2	Cytokine_receptors	2.362857	1.701484	3.28131
BPHL	Antimicrobials	0.637487	0.504375	0.80573
CLEC11A	Cytokines	1.516036	1.301901	1.765391
CMTM3	Cytokines	1.939969	1.499121	2.510458
CRIM1	Cytokine_receptors	1.923839	1.533551	2.413454
CTGF	Cytokines	1.603191	1.381789	1.860068
CYR61	Cytokines	1.384955	1.224587	1.566325
DAK	Antimicrobials	0.586742	0.442452	0.778088
DES	Antimicrobials	1.205936	1.13654	1.279568
EDNRA	Chemokine_receptors	1.499985	1.315628	1.710175
ELN	Antimicrobials	1.490388	1.305397	1.701595
ENG	Cytokine_receptors	1.391229	1.18348	1.635447
ESM1	Cytokines	1.462723	1.244433	1.719303
GNAI1	Antimicrobials	1.427925	1.196914	1.703522
GREM1	Cytokines	1.317087	1.181972	1.467647
GRP	Cytokines	1.227876	1.140799	1.321601
HDGFRP3	Cytokines	1.603254	1.380716	1.86166
IL1R1	Cytokine_receptors	1.613095	1.373481	1.894511
INHBB	TGFb_family_member	1.544374	1.362625	1.750365
LTBP3	Cytokines	1.722496	1.358221	2.184469
NOV	Cytokines	1.563551	1.372153	1.781646
NOX4	Antimicrobials	1.639326	1.41234	1.902793
NPR3	Cytokine_receptors	1.849908	1.547669	2.21117
NR2F1	Cytokine_receptors	1.378966	1.224325	1.55314
NRP1	Cytokine_receptors	1.707208	1.420286	2.052093
NRP2	Cytokine_receptors	1.691583	1.393531	2.053385
OSMR	Cytokine_receptors	1.74949	1.417618	2.159056
OXTR	Cytokine_receptors	1.405633	1.201078	1.645026
PDGFC	Cytokines	1.619596	1.361091	1.927198
PDGFRB	Cytokines	1.508678	1.304742	1.744491
PTGER3	Cytokine_receptors	1.630084	1.371071	1.938027
RBP1	Antimicrobials	1.486226	1.289005	1.713621
SDC2	Cytokine_receptors	1.822409	1.522803	2.180962
SEMA4C	Chemokines	2.225396	1.693848	2.92375
SHC4	NaturalKiller_cell_cytotoxicity	1.926967	1.615581	2.298371
SLIT2	Chemokines	1.469846	1.32759	1.627345
STC2	Cytokines	1.506128	1.26909	1.78744
TGFB2	Cytokines	2.056681	1.636983	2.583983
TGFB3	Cytokines	1.5918	1.342242	1.887756
THBS1	Antigen_processing_and_presentation	1.487585	1.310701	1.688341
TNC	Chemokines	1.286883	1.174997	1.409424
TPM2	Antimicrobials	1.441131	1.297227	1.600998

**Table 2:** Univariate and multivariate Cox regression analyses.

Univariate Cox		HR	HR.95L	HR.95H
Gender	Male/female	1.109543	0.870532	1.414176
Age	>65/≤65	1.312168	1.043428	1.650125
Stage	IV/III/II/I	2.339875	2.041174	2.682288
Lauren classification	Diffuse&mixed/intestinal	1.371609	1.092159	1.722562
Risk score	High/low	2.677984	2.102358	3.411215
Multivariate Cox				
Gender	Male/female	1.015188	0.793251	1.29922
Age	>65/≤65	1.541482	1.223368	1.942316
Stage	IV/III/II/I	2.284629	1.984801	2.62975
Lauren classification	Diffuse&mixed/intestinal	1.073366	0.85091	1.353981
Risk score	High/low	2.370156	1.850191	3.036248

## Data Availability

The datasets analyzed for this study were obtained from the Gene Expression Omnibus (https://www.ncbi.nlm.nih.gov/geo/), ImmPort database (https://immport.niaid.nih.gov), and The Cancer Genome Atlas (TCGA, https://portal.gdc.cancer.gov/).

## Abbreviations

GC: Gastric cancer
TCGA: The Cancer Genome Atlas
GEO: Gene Expression Omnibus
IRGs: Immune-related genes
LASSO: Least absolute shrinkage and selection operator
ROC: Receiver operating characteristic
AUC: Area under the curve
OS: Overall survival
RFS: Recurrence-free survival
ICI: Immune cell infiltration
ESTIMATE: Estimation of STromal and Immune cells in MAlignant Tumor tissues using Expression data
TME: Tumor microenvironment
GSEA: Gene set enrichment analysis
ES: Enrichment score.

## Supplementary Materials

Supplementary Materials Supplement material 1: the detailed clinical information of meta-GEO cohorts. Supplement material 2: the comparison of the proposed signature with others' related reports. Supplement material 3: the time-dependent ROC curves. Supplement material 4: AJE language editing certificate.
