# Supplementary material for: Development and Validation of a Robust Immune-Related Prognostic Signature for Gastric Cancer
Source: J Immunol Res. 2021 Apr 30;2021:5554342. doi: 10.1155/2021/5554342 (PMC8110424; doi:10.1155/2021/5554342)

Supplement material-1 The detailed clinical information of meta-GEO cohorts

|  | Patients with GC(n) |
| --- | --- |
| Project |  |
| GSE62254 | 300 |
| GSE15459 | 191 |
| GSE26901 | 109 |
| Survival status |  |
| alive | 298 |
| dead | 302 |
| Gender |  |
| female | 202 |
| male | 382 |
| Age |  |
| <=65 | 335 |
| >65 | 249 |
| Stage |  |
| I | 95 |
| II | 139 |
| III | 201 |
| IV | 149 |
| Lauren classification |  |
| Intestinal | 324 |
| Diffuse&Mixed | 260 |

Supplement material-2 The comparison of the proposed signature with others’ related reports

| Project | PMID: 33072557 | risk score |
| --- | --- | --- |
|  | AUC |  |
| GSE26253(Seoul,south Korea) |  |  |
| 3-year RFS | 0.565 | 0.616 |
| 4-year RFS | 0.576 | 0.625 |
| 5-year RFS | 0.562 | 0.618 |
| GSE26901(Busan, Korea) |  |  |
| 3-year OS | 0.646 | 0.735 |
| 4-year OS | 0.631 | 0.737 |
| 5-year OS | 0.634 | 0.755 |
| GSE62254(Asian Cancer Research Group) |  |  |
| 3-year OS | 0.615 | 0.715 |
| 4-year OS | 0.613 | 0.71 |
| 5-year OS | 0.604 | 0.703 |
| GSE84437(South Korea) |  |  |
| 3-year OS |  | 0.612 |
| 4-year OS |  | 0.622 |
| 5-year OS |  | 0.625 |
| GSE15459(Singapore Patient Cohort) |  |  |
| 3-year OS |  | 0.756 |
| 4-year OS |  | 0.766 |
| 5-year OS |  | 0.772 |
| TCGA(white, asian,black,etc) |  |  |
| 3-year OS | 0.694 | 0.625 |
| 4-year OS | 0.706 | 0.614 |
| 5-year OS | 0.674 | 0.658 |
| TCGA-Asian |  |  |
| 1-year OS |  | 0.711 |
| 2-year OS |  | 0.715 |
| 3-year OS |  | 0.741 |
| 4-year OS |  | 0.741 |

Supplement material-3 The time-dependent ROC curves


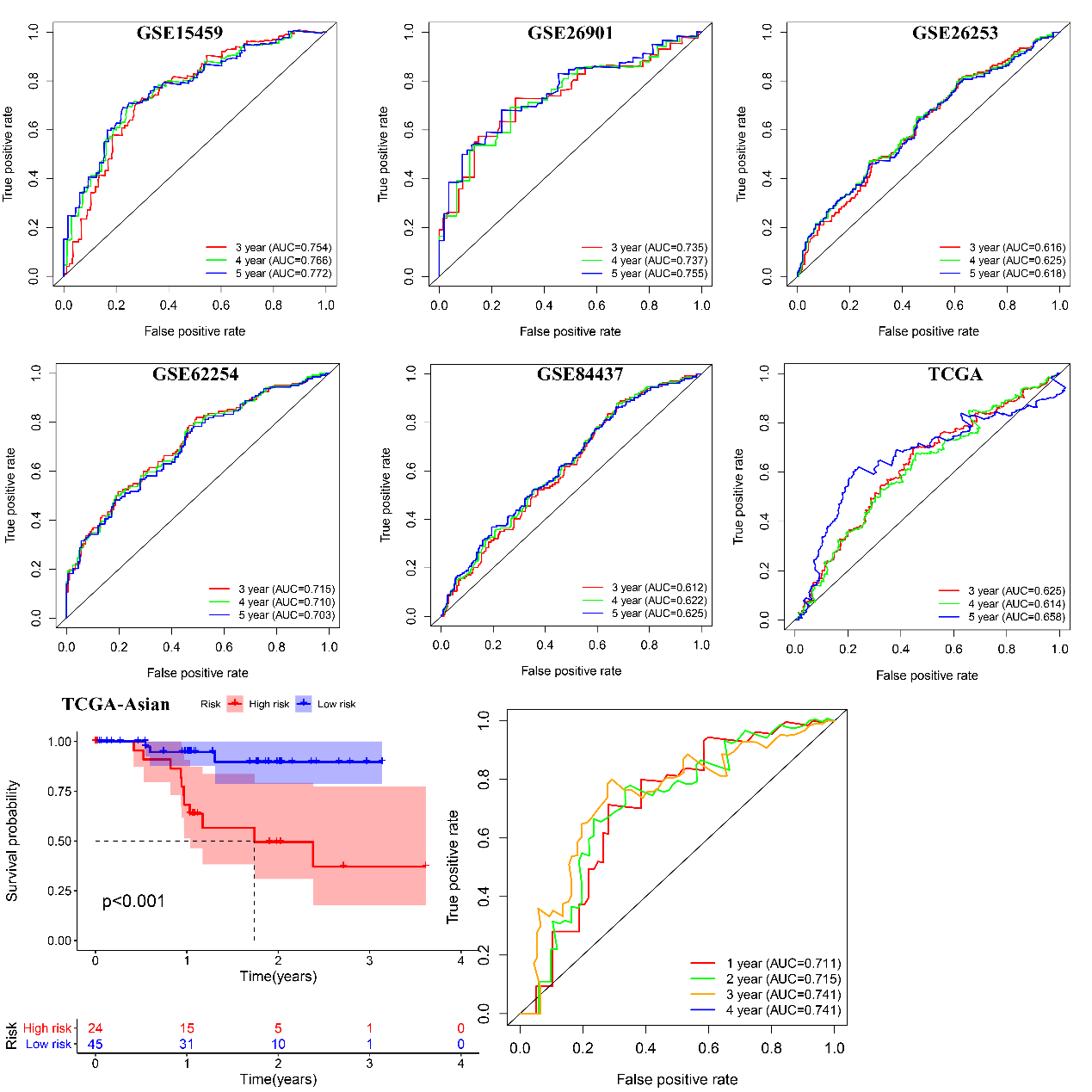


Supplement material-4 AJE language editing certificate


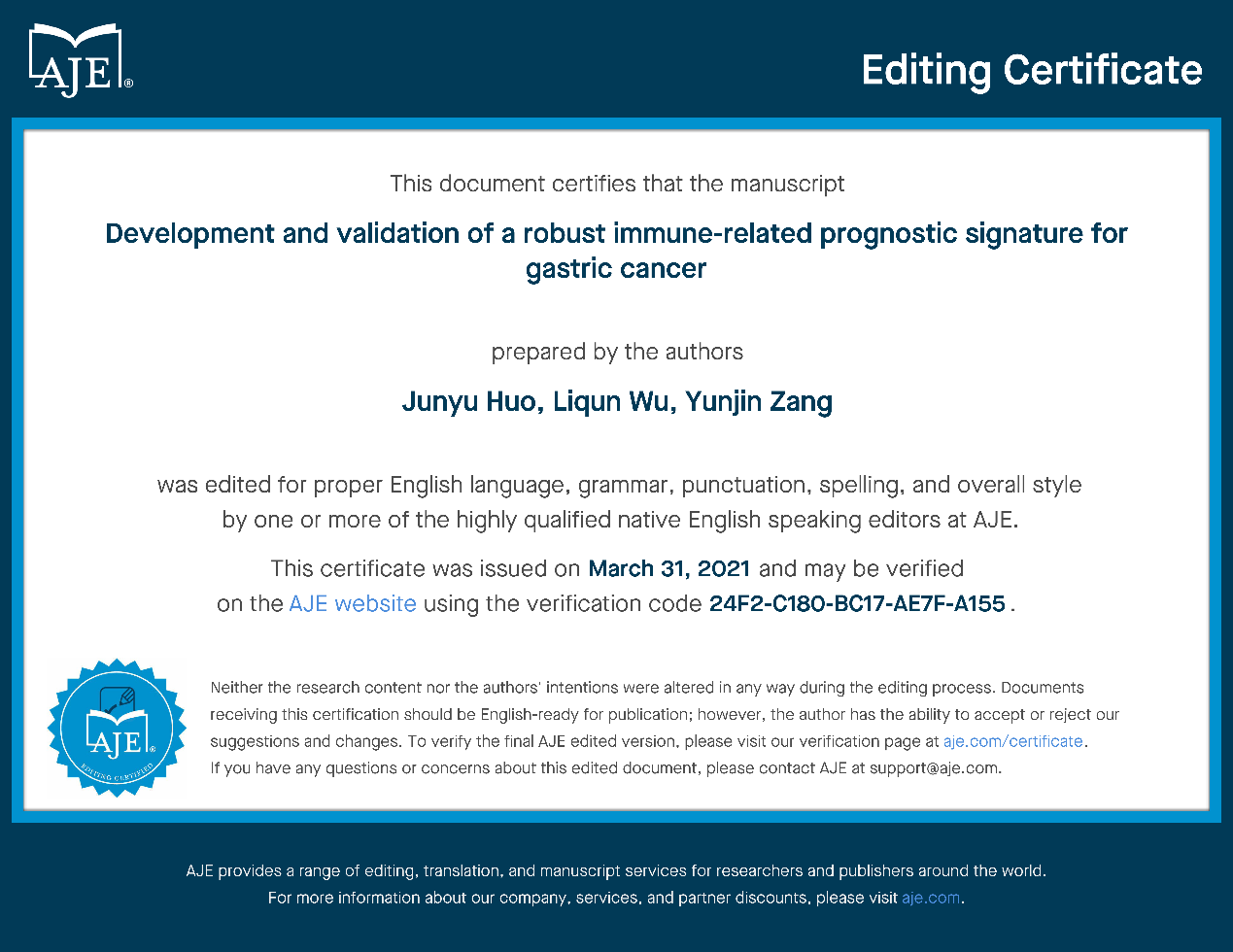

Supplement: Supplementary Materials — Supplement material 1: the detailed clinical information of meta-GEO cohorts. Supplement material 2: the comparison of the proposed signature with others' related reports. Supplement material 3: the time-dependent ROC curves. Supplement material 4: AJE language editing certificate. [file 5554342.f1.docx]
